# Forming of Components with Microgearings from Coil Material—Numerical Modeling of the Process Chain and Experimental Validation

**DOI:** 10.3390/mi12121456

**Published:** 2021-11-26

**Authors:** Andreas Rohrmoser, Martin Kraus, Marion Merklein

**Affiliations:** Department of Mechanical Engineering, Institute of Manufacturing Technology, Friedrich-Alexander-Universität Erlangen-Nürnberg, Egerlandstr. 13, 91058 Erlangen, Germany; martin.kraus@fau.de (M.K.); Marion.Merklein@fau.de (M.M.)

**Keywords:** micro forming, microgear, sheet-bulk metal forming, multi-step micro forming

## Abstract

Compared to alternative production methods, cold forming offers technological, economic and ecological potential for the mass production of microgears. Within the current boundaries of the technology, the cold forming of modules m < 0.2 mm is not possible due to size effects, high tool stresses and handling problems. The investigations of this contribution present a novel process chain for the multi-step forming of microgears with a module of m = 0.1 mm. For this purpose, a numerical model of the first two steps of the process chain is set up and confirmed based on experimental forming tests. The results have proven the feasibility of the process chain by a complete forming of the gear teeth.

## 1. Introduction

One trend in today’s industry is the miniaturization of technical systems with electronic and mechanical functions. This includes miniaturized drive systems which are used in large numbers; for example, in the automotive industry, mechanical engineering, aerospace technology, medical technology, the watch industry and in robotics [1]. Due to high production volumes, the use of productive manufacturing processes is of great economic importance. At present, the production of microgears is completed industrially by primary shaping via metal powder injection molding, cutting by means of fine cutting, spark erosion, laser ablation, honing, milling, broaching, grinding, shaping and additive processes [2]. However, these technologies have process-specific limitations. Micro metal injection molding (µ MIM) requires a complex process chain, and the achievable part density and surface quality are primarily limited by the particle size of the used powder [3]. Moreover, long process times reduce the application in mass production [4]. With cutting, the obtainable gear width is restricted, and for hobbing processes producible geometries are limited and no internal gears or conical gears can be produced [1].

Compared to established processes, the cold forming of microgears offers great potential. In macroscopic size mass production of geared metallic parts, cold forming has technological, economic and ecological advantages: high volume output with short cycle times; improved component properties due to strain hardening in the tooth root and flank area; a ductile core and an uninterrupted fiber flow; and a high surface quality are achieved [5]. However, the application in micro scale is limited due to the high stresses it places on small gear cavities [2] and occurring tool wear [1], as well as size effects. The lower module limit for the use of cold forming for the production of microgears is at a module of m < 0.2 mm, according to the review of Jain and Chaubey [1]. In [6], Ganji et al. successfully produced microgears from CU-OFE with a modulus of 0.25 mm. Chen et al. investigated a two-stage process, which was divided into upsetting and forging [7]. Double gears with moduli of 0.12 mm and 0.15 mm were formed by this hybrid process, where the mold filling was limited as the gear width increased [7].

During forward extrusion of gears, critical tensile stresses occur in the area of the die teeth [8]. The high stress on the small tips of the gearing leads to wear, resulting in a decrease in the quality of the components as well as potential tool failure [2]. To achieve a reduction of the producible module by cold forming, it is necessary to reduce the tensile stresses. Furthermore, there are challenges with regard to incomplete die filling and inhomogeneous shaping when forming micro geometries by polycrystalline metals due to the forming mechanisms of grain boundary sliding and grain rotation [9]. According to Saotome and Xu, the die filling is improved by decreasing the grain size [9]. A homogeneous forming can be achieved by using nanocrystalline microstructures [10].

A promising approach to meet these challenges is offered by micro bulk forming geared components from coil in a multi-step forming process. Within this contribution, a novel process chain is presented. In the first step, a pin is extruded from the sheet metal plane in order to provide material for the second process step. This kind of pin extrusion was first introduced by Hirota [11]. In the second stage of the investigated process chain, upsetting and lateral extrusion are used to form the gear teeth. Previous investigations on the production of filigree functional elements by sheet-bulk metal forming show that significantly lower die stresses occur during lateral extrusion than during full forward extrusion [12]. In addition, through the approach of multi-step forming, a high degree of grain deformation is achieved, which results in grain refinement and may improve die filling. After microgear forming, the gear can be separated from the sheet by means of shear cutting. In addition to reduced tool stresses, the process chain offers the potential to achieve simplified handling of micro components and high productivity through the use of coil material.

## 2. Objective and Methodology

The objective of this investigation is the development and validation of a numerical process model of the described process chain, as well as proof of the feasibility of the process. Possible size effects are assessed with the help of geometric measurements and metallographic examinations. Subsequently, the validated FE model is used to further analyse the material flow and process force progression. This provides the basis for additional research. A high model accuracy is achieved by characterizing the anisotropic flow behaviour and determining the tribological conditions by laboratory tests.

## 3. Process Layout

**Figure 1:**
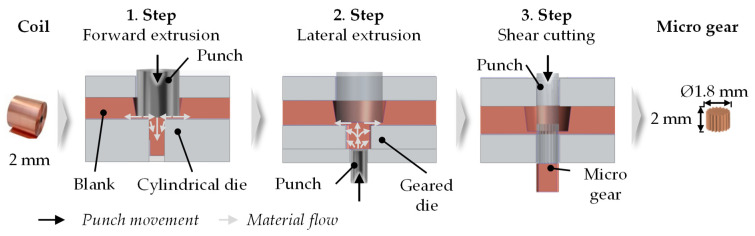
Multi-step forming process for microgear production.

The investigated microgear had an involute profile with a normal module of m = 0.1 mm and a root radius of 0.06 mm. The tip diameter was 1.8 mm and the root diameter was 1.55 mm. The gear width was 2 mm. Figure 1 shows the applied novel process chain.

In the first process step, full forward extrusion was used to form a pin from the sheet metal plane and to provide material for the manufacturing of the gearing in the second process step. The diameter of the pin was 1.5 mm. In the second process step, the gearing is formed from the pin by lateral extrusion. The extrusion dies were made of submicron grain-cemented carbide (CFS18Z) and reinforced according to VDI 3176 with a pre-stress ring with 5‰ excess. During both processes, a blank holder with a pressure of 15.6 MPa, which represents 99% of the material’s initial yield stress, was used to ensure no plastic deformation by the blank holder.

Due to its high purity and single-phase grain structure, the copper material Cu-OFE is excellently suited to investigate size effects. A uniformly coarse grain structure was achieved without the influence of the rolling process of the sheet by soft annealing the material at 650 °C for 1 h under an inert gas atmosphere. In order to simplify the experimental setup and the conduction of the forming tests, the investigations were carried out on separate circular blanks instead of strip material. The geometry of the sheet material as well as the forward extrusion punch were chosen based on preliminary numerical studies. The diameter of the blank was 20 mm with a thickness of 2 mm. They were made by water jet cutting to avoid heat influence. A punch with a diameter of 4 mm was used during full forward extrusion. *Dionol ST V 1725-2* impact extrusion oil from *MKU-Chemie GmbH* was used as a lubricant in a quantity of more than 10 g/m^2^.

## 4. Numerical Process Model and Experimental Validation

**Figure 2:**
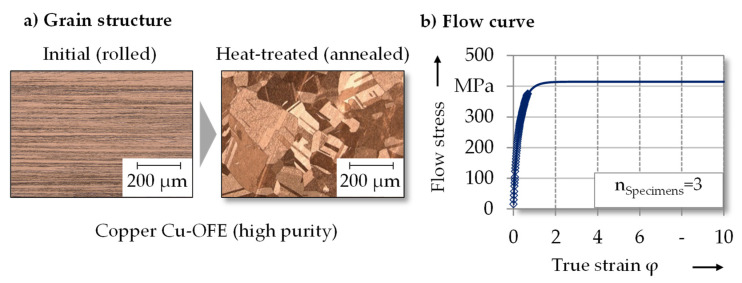
(**a**) Grain structure and (**b**) flow curve determined by tensile tests 0° to the rolling direction and extrapolated with the Hockett-Sherby approach.

Figure 2a shows the grain structure and flow curve of the gear material. The grain structure was made visible by metallographic preparation and etching with ammonium peroxodisulfate. The heat-treated material was coarse-grained with an average grain diameter of 125 µm according to ISO 643.

The flow curve (Figure 2b) was determined by uniaxial tensile tests in rolling direction (0°) up to a true strain of φ = 0.25. The experimentally determined flow curve was extrapolated to map higher strains as they occurred during gear extrusion with the Hockett-Sherby approach [13]. The anisotropic flow behavior was determined by uniaxial tensile tests in the 0°, 45° and 90° directions according to ISO 6892-1 and hydraulic bulge tests (ISO 16808). Material anisotropy was considered in the simulation using the yield function Barlat91 [14]. Due to comparable contact pressures, the tribological conditions were evaluated by single sheet metal compression tests within a prior investigation [15]. The corresponding friction factor m = 0.045 ± 0.012 was determined by sheet metal tests and numerical identification. The FE software simufact.forming 16.0 was used within the investigation. In order to reduce calculation effort, a 90° segmented model was applied. This enabled the consideration of planar anisotropy. The workpiece was meshed with hexahedrons to accurately map the three-dimensional material flow. During first process step, the center area of the workpiece as well as the area with reduced sheet thickness was meshed with a minimum edge length of 40 µm. In the outer area of the sheet blank, the mesh was coarsened up to 100 µm edge length in order to reduce the element number. Likewise, in the second process step, the geared area was more finely meshed than the remaining area with an element size of 40 µm. The workpiece was remeshed if the elongation exceeds 40%. The calculation of both process steps was divided into 1000 increments and solved with the multifrontal method.

**Figure 3:**
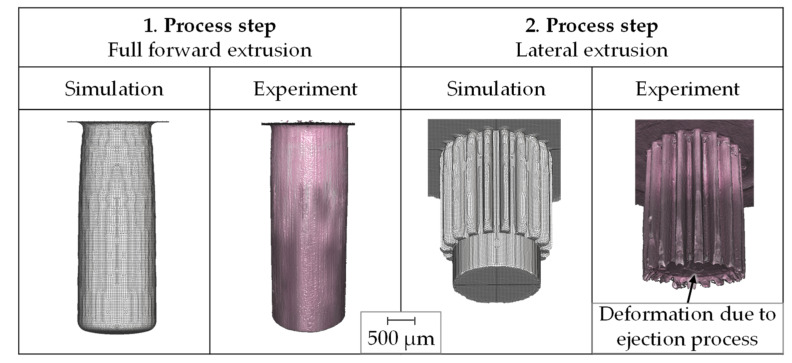
Numerically and experimentally determined geometries.

After the process chain had been modeled and designed numerically, it was implemented in forming tests. Based on these, the numerical model aws validated and possible size effects that could not be represented in the simulation were identified. The forming tests were carried out on a universal testing machine *walter+bai FS-300*. A total number of 30 gears were produced. The measurement of the component geometry and surface topography was performed with the 3D measurement system *Alicona InfiniteFocus XL 200 G5* by focus variation. Figure 3 shows the numerical and experimental components after both process steps.

In addition to the lateral extrusion, an ejection process was carried out in the experiment, which was not taken into account in the simulation. Due to the high force required to eject the gearing (max. 2 ± 0.3 kN), deformation of the cylindrical shoulder occurred. While the component was pushed upwards through the geared die, the cylindrical shoulder was therefore also formed into the gear geometry.

**Figure 4:**
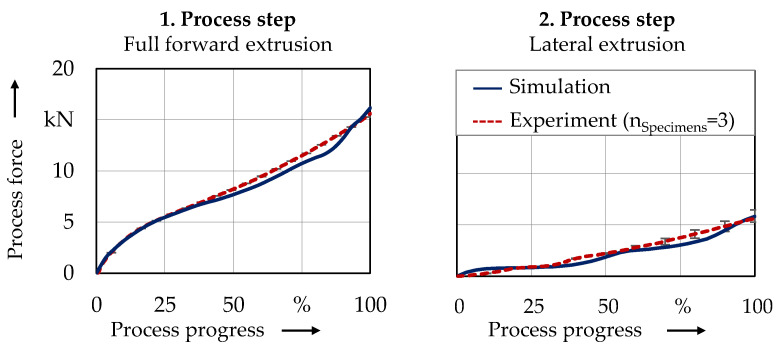
Comparison of force curve progression in simulation and experiment.

The numerically determined process properties were validated based on the force curve progression and the component geometry. Figure 4 shows the process force curves from simulation and experiment.

**Figure 5:**
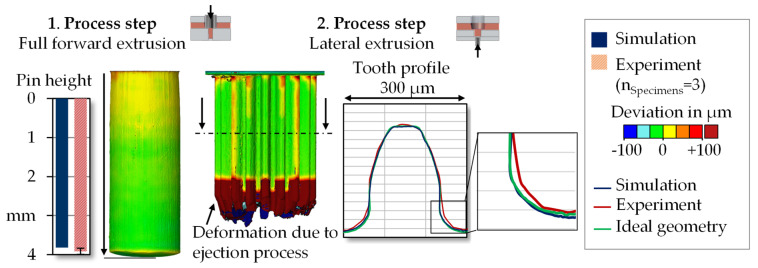
Comparison of component geometry in simulation and experiment.

The process force curves show good agreement. The maximum process forces required for forward extrusion (16.2 kN simulation and 15.6 ± 0.7 kN experiment), as well as for lateral extrusion (7.0 kN simulation and 6.6 ± 0.5 kN experiment) deviate less than 6%. A comparison of the geometric properties is made in Figure 5. Moreover, regarding the die filling, the simulation showed a good agreement.

The pin heights differed slightly, being 3.8 mm in the simulation and 4.2 ± 0.1 mm in the experiment. With regard to the gearing formed in the second process step, there is good agreement between experiment and simulation for the majority of the gear width. In the lower area of the workpiece, however, there was a deformation of the cylindrical shoulder due to the ejector process, which was not taken into account in the simulation. The evaluation of the tooth profiles in the face section showed a very high form filling of the experimentally produced gears with minimal deviation from the ideal geometry. It is noticeable that the experimentally determined tooth geometry showed a slight material excess in the tooth root area. Reasons for this could be manufacturing-related deviations as well as the elastic deflection of the die, which were not considered in the simulation. The surface roughness of the die could not be measured due to limited accessibility, but the surface roughness of an external gearing manufactured with the same electrical discharge machining process was Rz 0.604 ± 0.165 µm. The surface roughness depth of the pin was R_Z_ 1.7 ± 0.2 µm, the roughness depth of the tooth flanks R_Z_ 1.8 ± 0.3 µm. Thus, a ready-to-use surface quality was achieved.

**Figure 6:**
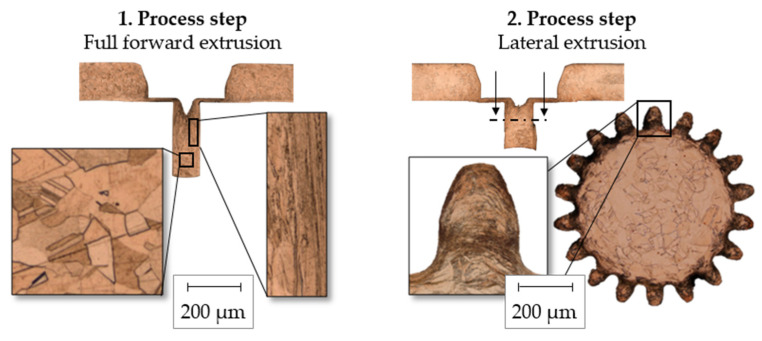
Resulting grain structure.

Figure 6 shows the resulting grain structure after both process steps. As seen in Figure 5, no problems due to size effects [9] in the shaping of the micro teeth (m = 0.1 mm) could be detected, despite the large grains in the initial grain structure. This is attributed to the grain refinement within the multi-step process route.

After the first process step, the grains were elongated and compressed in the edge area of the pin in which the toothing was later formed. During lateral extrusion, the grain structure was further refined and the elongated grains were aligned along the tooth profile. These grains are small enough to fill the micro gearing (m = 0.1 mm) completely. Due to the high agreement of the experiment regarding process force and die filling with the FE model, significant influences of size effects in the process chain could be excluded.

## 5. Analysis of the Material Flow

**Figure 7:**
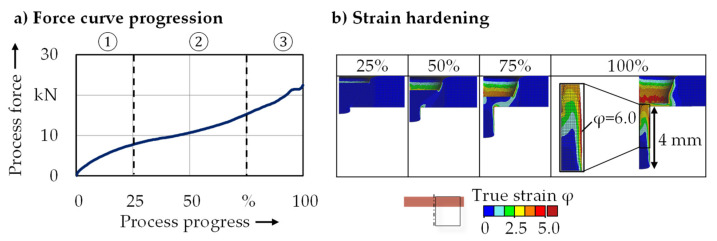
(**a**) Force curve progression and (**b**) strain hardening during full forward extrusion.

In the following, the validated FE model will be used for a detailed analysis of the material flow, in order to enhance the process understanding. Figure 7 shows the progression of the process force and material flow during full forward extrusion. Three distinct process phases can be identified in the force progression (Figure 7a), which have already been described by Ghassemali et al. [16]. At the beginning of the process (1), elastic then plastic deformation occurred when the punch moved downwards and contacted the sheet metal (1). After that, the sheet was further compressed and the pin was shaped while forming force rose as a result of increased wall friction and material hardening (2). Towards the end of the process (3), as the thickness of the remaining sheet decreased, the process force increased up to a maximum of 16.2 kN. The highest degree of deformation (φ = 6.0) arose at the lateral surface of the pin (Figure 7b) where the gearing would subsequently be formed and grain refinement was observed (Figure 6).

**Figure 8:**
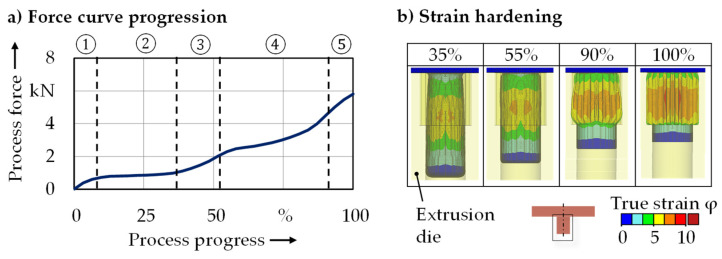
(**a**) Force curve progression and (**b**) strain hardening during lateral extrusion.

After the first process step, the extruded pin was positioned in the geared die for lateral extrusion. Based on the process force curve and the material flow, different phases could be identified (Figure 8a).

Initially, there was upsetting of the pin (1), until the pin made first contact with the cylindrical area of the die (at about 8%). The further upsetting process took place due to the decreasing pin height with declining wall friction and increasing upsetting force, resulting in a constant force requirement (2). At about 35% process progress, the diameter of the pin reached the root diameter of the toothing, leading to an elevated forming force due to the increased deformation resistance as a result of wall friction rising again (3). After the pin was in complete contact with the root diameter of the die, the forming of the gearing began. The force requirement rose as the gearing was filled and wall friction increased (4). Towards the end (5), the force ascended to a maximum of 7.0 kN due to maximum wall friction and the influence of the increasing work hardening of the material. The force level in lateral extrusion was significantly lower than that of pin extrusion. Since the volume of the pin was greater than the volume of the gear, a cylindrical shoulder remained in the lower area (Figure 8b).

## 6. Summary and Outlook

Within this contribution, a numerical process model of a novel multi-step forming process for the manufacturing of microgears from sheet metal was built. The feasibility of the process chain was proven and the prediction quality of the FE model was validated, followed by a detailed analysis of the material flow. The gears produced with a module of m = 0.1 mm represent an enhancement beyond the current limits of the cold forming of microgears. No size effects regarding die filling were determined and complete tooth filling was achieved. In addition, further tests with samples of different grain sizes should be carried out for a better understanding of the influence of size effects.

There is need for additional research with regard to the further development of the process chain. The forming tests have shown that the ejection process after lateral extrusion leads to additional deformation of the cylindrical area and must be taken into account. Against this background, i.e., that the forming of the microgears from the coarse-grained copper material was successful, other relevant gear materials such as brass or steel should be investigated.

Particular potential is seen in the industrial implementation of the process chain in a multi-step serial production starting from coil material. However, this requires the realization of the third process step by separating the gear from the remaining sheet. Since the residual sheet thickness is only 0.2 mm, shear cutting is feasible according to the current state of the technology.
